# The impact of Movement Control Order during the COVID-19 pandemic on lifestyle behaviours and body weight changes: Findings from the MyNutriLifeCOVID-19 online survey

**DOI:** 10.1371/journal.pone.0262332

**Published:** 2022-01-18

**Authors:** Yit Siew Chin, Fui Chee Woon, Yoke Mun Chan

**Affiliations:** 1 Department of Nutrition, Faculty of Medicine and Health Sciences, Universiti Putra Malaysia, Seri Kembangan, Selangor, Malaysia; 2 Research Centre of Excellence, Nutrition and Non-communicable Diseases, Faculty of Medicine and Health Sciences, Universiti Putra Malaysia, Seri Kembangan, Selangor, Malaysia; 3 Department of Dietetics, Faculty of Medicine and Health Sciences, Universiti Putra Malaysia, Seri Kembangan, Selangor, Malaysia; Kasturba Medical College Mangalore, INDIA

## Abstract

**Background:**

The COVID-19 pandemic lockdowns have affected daily lives of the communities worldwide. This study aims to determine the lifestyle behaviours and their associations with body weight changes among Malaysian adults during the Movement Control Order (MCO) due to COVID-19 pandemic.

**Methods:**

A total of 1319 Malaysian adults participated in this cross-sectional online survey. Information on anthropometric data including body weight and height, and lifestyle behaviours including eating pattern, physical activity, and sleep pattern were self-reported by the respondents. A multivariable generalised linear mixed model was used to assess the associations between lifestyle behaviours and body weight changes with adjustment of confounding factors; namely, age, sex, ethnicity, and body weight status before MCO.

**Results:**

During MCO, 41.2% of the respondents perceived that their eating patterns were healthier, but 36.3% reduced their physical activities, and 25.7% had a poorer sleep quality. Further, the proportion of adults who reported having lose weight (32.2%) was almost similar to those who reported having gained weight (30.7%). Lifestyle behaviours including less frequent practice of healthy cooking methods and lunch skipping were associated with weight gain, while less frequent consumption of high fat foods, more frequent physical activity, and good sleep latency were associated with lower risk of weight gain. In contrast, practicing healthy eating concept, skipped lunch, and more frequent physical activity were significantly associated with weight loss.

**Conclusion:**

Lifestyle behaviours were associated with body weight changes during MCO. While the COVID-19 pandemic lockdown is necessary to prevent further spread of the disease, promoting healthy lifestyle practices during lockdown should be implemented for a healthy weight and better health.

## Introduction

The coronavirus disease 2019 (COVID-19), caused by a novel coronavirus, SARS-CoV-2 was first reported in December 2019 in Wuhan of China [1]. The disease has quickly spread throughout the world and was declared as a global pandemic on March 11, 2020 by the World Health Organization (WHO) [2]. Mounting evidence show that obesity is a major risk factor for severe COVID-19 infection [3, 4], with obese individuals demonstrated a higher risk for COVID-19 positive, hospitalisation, intensive care unit (ICU) admission, and mortality [5–8].

The first COVID-19 case in Malaysia was recorded on January 25, 2020 and increased to 673 cases with two deaths on March 17, 2020 [9, 10]. On March 18, 2020, the Malaysian government announced a national lockdown, also known as the Movement Control Order (MCO), due to persistent increase in new COVID-19 cases and the MCO was extended to May 4, 2020 [9]. During the MCO, mass movements and gatherings at all places are prohibited, all business premises are closed except for those selling daily necessities and learning institutions of all types are closed [9].

Although the lockdown is necessary to prevent further spread of the disease, there are reasons to be concerned because prolonged home confinement during a disease outbreak could lead to dramatic changes in lifestyle behaviours of the population and subsequent changes in body weight [11]. Early study showed that home confinement is associated with changes in food consumption and meal patterns, lower levels of physical activity, and increased sedentary behaviours [12–14]. In addition, continuously hearing or reading about the pandemic without a break can be stressful during home confinement, contributed to over- or under-eating [15–17] and negative emotions such as fear and anxiety that have negative impacts on overall sleep quality [18]. All the abovementioned lifestyle behavioural changes may lead to significant changes in body weight [11–14].

There is limited evidence to determine the effects of COVID-19 pandemic lockdown on body weight changes [11–14]. So far, there is no data with respect to the changes in lifestyle behaviours during the lockdown and their associations with changes in body weight among Malaysian adults. Therefore, MyNutriLifeCOVID-19 study was conducted to determine the lifestyle behaviours during the lockdown and to assess whether these lifestyle behaviours are associated with body weight changes.

## Materials and methods

### Study design and respondents

This study is a cross-sectional online survey conducted among Malaysian adults aged 18 years and above, which is known as MyNutriLifeCOVID-19 survey. The survey was conducted between April 21 and June 7, 2020 using the Google online survey platform. In Malaysia, the MCO period was enforced between March 18 and May 4, 2020. After May 4, 2020, a conditional MCO (CMCO) was implemented, which allowed conditional resumption of specific economic and social sectors to ease economic losses in the country. The link to the online survey was disseminated through e-mails, social media such as Facebook, Instagram, and WhatsApp, as well as personal networks of the respondents. The study was conducted in agreement with the Declaration of Helsinki and the protocol was approved by the Ethics Committee for Research Involving Human Subjects of Universiti Putra Malaysia (JKEUPM-2020-163). At the beginning of the online survey, information on study background, objectives, and scope of questions asked were provided. Statements describing that participation is voluntary, and that participant may withdraw anytime without penalty or loss of benefit to which the participant is entitled were made available on the google form, before the participants agreed and gave their written consent and continue with the online survey. The participants were also informed that all data collected would be used for research purposes only and their permission for data sharing and publication was obtained before answering the online survey.

### Questionnaires

The self-administered questionnaire consisted of five sections that assessed socio-demographic characteristics, body weight status, disease history, and lifestyle habits that include eating pattern, physical activity and sleep quality. The questionnaire was available in three languages including English, Malay, and Chinese. The original English language questionnaire was translated into Malay and Chinese, respectively, and back-translated by university lecturers with health sciences background and proficient in all the three languages. Both translated and back-translated questionnaires were compared for consistency. The questionnaire was then pre-tested prior to data collection to ensure clarity and ease of understanding of the questionnaire by the respondents.

#### Characteristics of the respondents

Information on socio-demographic characteristics including age, ethnicity, sex, educational level, marital status, occupation, monthly household income, number of family members, and current living condition were collected. Self-reported history of diseases and type of diseases were assessed. Meanwhile, adoption of any weight management strategies during MCO were ascertained.

#### Anthropometric information

Information on height, current body weight, and last known weight before MCO were self-reported by the respondents. Body mass index (BMI) was calculated by dividing the weight in kilograms with the square of height in meters and classified into underweight (< 18.5 kg/m2), normal weight (18.5–24.9 kg/m2), overweight (25.0–29.9 kg/m2), and obesity (≥ 30.0 kg/m2) according to WHO classifications [19]. Body weight changes were calculated as the difference between current body weight and the last known weight before MCO and categorised as weight decreased, no difference, or weight increased. Self-reported weight and height are commonly used in large epidemiological studies and evidence has demonstrated good agreement between self-reported and direct anthropometric measurements [20–22].

#### Eating pattern

A series of self-developed questions aims to identify the changes of eating pattern during MCO was administered by the respondents. These include (1) perceived eating behaviours changes during MCO in comparison to pre-MCO (response options were “less healthy”, “no difference”, and “healthier”); (2) dietary habits including consuming homecooked meals, consuming foods or drinks from restaurants/hawker centres/coffee shops/other food stalls, consuming foods or drinks from western fast food restaurants, going out to pack foods/drinks, ordering foods/drinks through Food Delivery Apps, obtaining free/donated foods/drinks, obtaining free foods/drinks, baking and preparing desserts at home, practicing healthier cooking methods, and practicing healthy eating concept “Quarter-Quarter-Half” (4-point Likert scale ranging from “never" to “everyday”); (3) Food group consumption including rice/noodles/bread/cereals/cereal products/tubers, egg/fish/chicken, meat and meat products, legumes and nuts, milk and dairy products, fruits, vegetables, sugar-sweetened beverages, fried foods/high fat foods, sweet foods/high sugary foods, dietary supplements, probiotic drinks (4-point Likert scale ranging from “never" to “everyday”); (4) Main meal consumption including breakfast, lunch and dinner as well as snacking between main meal consumption (4-point Likert scale: “(1) everyday; (2) 4–6 times/week; (3) 1–3 times/week; and (4) never”).

#### Physical activity

Physical activity was assessed by asking if the respondents were performing any physical activities or exercise for at least 30 minutes per day during MCO and rated on a 5-point Likert scale: (1) everyday; (2) 5–6 days/week (3) 3–4 days/week; (4) 1–2 days/week; and (5) never. The 5-point Likert scale was re-categorised into (1) <5 days/week and (2) ≥5 days/week for analysis of this variable in accordance with the Malaysian Dietary Guidelines 2010 [23] that recommends “at least 30 minutes moderate intensity physical activity on at least 5–6 days a week”. Meanwhile, respondents were asked if there are any changes in the pattern of exercise or physical activity they performed during MCO as compared to pre-MCO and rated on a 4-point Likert scale: (1) never exercise; (2) less; (3) as usual; and (4) more”.

#### Sleep pattern

Sleep pattern including actual sleep duration, sleep latency, and overall sleep quality were measured using the Pittsburgh Sleep Quality Index (PSQI) [24]. Actual sleep duration was assessed by asking the respondents “During the past month, how many hours of actual sleep did you get at night?” with response options categorised into “(1) ≥7 hours; (2) 6–7 hours; (3) 5–6 hours; and (4) <5 hours”. Sleep latency was assessed by asking two questions; namely, “(1) During the past month, how long has it usually take you to fall asleep each night?” with response categorised into “(0) ≤15 minutes; (1) 16–30 minutes; (2) 31–60 minutes; and (3) >60 minutes” and “(2) During the past month, how often have you had trouble sleeping because you cannot get to sleep within 30 minutes” with response options categorised into “(0) Not during the past month; (1) Less than once a week; (2) Once or twice a week; and (3) Three or more times a week”. Scores for the two questions were summed to obtain a total score. The total score ranges from 0–6 and categorised into “(0) Very poor; (1) Poor; (2) Average; (3) Good”. Overall sleep quality was assessed by asking: “During the past month, how would you rate your sleep quality overall?” and rated on a 4-point Likert scale: “(1) very good; (2) fairly good; (3) fairy bad; (4) very bad”. In addition, respondents were asked if there were any changes in their sleep quality during MCO as compared to pre-MCO and rated on a 3-point Likert scale: (1) better; (2) o difference; (3) poorer”.

### Statistical analysis

Data was analysed using IBM SPSS Statistics 24 software (SPSS Inc., Chicago, IL, USA). Descriptive statistics were presented as frequency and percentage for categorical variables while mean and standard deviation for continuous variables. The chi-square test of independence was used to determine the bivariate associations between the lifestyle behaviours and body weight changes. Upon completion of the bivariate analysis, variables with a p-value < 0.05 were included in the multivariable generalised linear mixed model to determine the associations between lifestyle behaviours and body weight changes during MCO. Study sites and respondents were entered as random effects. Multivariable models were adjusted for potential confounding variables including age, sex, ethnicity, and BMI categories before MCO. Data were presented as odds ratio (OR) and 95% confidence interval (CI). All statistical significance level was set at p < 0.05.

## Results

### Characteristics of the respondents

Characteristics of the study respondents are shown in Table 1. A total of 1319 Malaysian adults participated in the present study with a mean age of 36.3 ± 11.2 years. Majority of them were females (76.3%), attained tertiary education (90.9%), had a moderate to high monthly household income (84.5%), and lived with their family members during MCO (79.2%). A quarter of them were Malays (44.4%), 51.9% were married, and more than half of them began working from home during MCO (54.3%). Less than one quarter of the respondents had chronic diseases (21.4%), with hypertension (8.5%), diabetes (5.2%), and hyperlipidemia (2.3%) as the top three common chronic diseases.

**Table 1:** Characteristics of the respondents.

Variables		All (n = 1319) n (%)
Age (years) ^a^		36.3 ± 11.2
Sex	Female	1007 (76.3)
	Male	312 (23.7)
Ethnicity	Malay	586 (44.4)
	Chinese	561 (42.5)
	Indian	98 (7.4)
	Others	74 (5.6)
Marital status	Married	685 (51.9)
	Single	593 (45.0)
	Divorced/Widowed	41 (3.1)
Highest educational level	Tertiary	1199 (90.9)
	Secondary	116 (8.8)
	Primary	4 (0.3)
Employment status during MCO	Began remote work	716 (54.3)
	Go to work as usual	157 (11.9)
	Mix mode	17 (1.3)
	Unable to work due to the pandemic	117 (8.9)
	A student	211 (16.0)
	Retired	31 (2.4)
	Unemployed	70 (5.3)
Monthly household income ^b^	Low (<RM2300)	205 (15.5)
	Moderate (<RM5600)	517 (39.2)
	High (≥RM5600)	597 (45.3)
Current living condition	Live alone	203 (15.4)
	With family	1045 (79.2)
	With friends	71 (5.4)
Number of family members ^a^		4.4 ± 2.3
Disease history	Healthy	1037 (78.6)
	With chronic disease	282 (21.4)

Abbreviations: RM, Ringgit Malaysia (1 USD = RM 4.15, as of May 20, 2021).

^a^ Data are presented as mean ± standard deviation (SD).

^b^ Classified according to The Economic Planning Unit [25].

### Changes of body weight and BMI category during MCO

Prior to MCO, about half of the respondents had a normal weight (54.7%), 7.8% were underweight, 25.5% were overweight, and 12.1% were obese. Table 2 shows the changes of body weight of the respondents during MCO. About one-third of the respondents gained weight during MCO (30.7%) with an average weight gain of 2.1 kg, while 32.2% lose weight with an average weight loss of 2.3 kg. About 11.0% of the respondents who were underweight before MCO reduced their body weight, while 46.3% gained weight, respectively. On the other hand, 29.4% of the respondents who were normal weight lose their weight, while 30.1% had an increased weight during MCO. More than one-third of the respondents who were overweight reduced their body weight (36.1%), while 29.4% gained weight during MCO. Meanwhile, half of those who were obese reduced their body weight (50.3%), while 25.7% gained weight during MCO. In terms of BMI category changes, 14.8% of the respondents who were underweight and 9.5% who were overweight attained normal BMI during MCO. For respondents who were normal weight before MCO, 1.5% and 4.5% of them became underweight and overweight, respectively. While 4.6% of the respondents who were overweight changed their BMI category to obesity during MCO, 13.8% of them who were obese changed their BMI category to overweight.

**Table 2:** Changes of body weight and BMI category during MCO.

Variables	Total (n = 1319)	BMI before MCO, n (%)				
		Underweight (n = 108)	Normal (n = 717)	Overweight (n = 328)	Obesity (n = 166)	*p*-value
**Body weight changes during MCO (kg)** ^**a**^	-0.1 ± 2.1	0.6 ± 1.3	0.0 ± 1.8	-0.3 ± 2.3	-0.9 ± 3.1	< 0.001
Decreased	425 (32.2)	12 (11.1)	211 (29.4)	118 (36.1)	84 (50.3)	< 0.001
No change	489 (37.1)	46 (42.6)	290 (40.4)	113 (34.6)	40 (24.0)	
Increased	405 (30.7)	50 (46.3)	216 (30.1)	96 (29.4)	43 (25.7)	
**BMI during MCO**						
Underweight	103 (7.8)	92 (85.2)	11 (1.5)	0	0	< 0.001
Normal weight	721 (54.7)	16 (14.8)	674 (94.0)	31 (9.5)	0	
Overweight	336 (25.5)	0	32 (4.5)	281 (85.9)	23 (13.8)	
Obesity	159 (12.1)	0	0	15 (4.6)	144 (86.2)	

^a^ Data are presented as mean ± standard deviation (SD).

### Lifestyle behavioural changes during MCO

As shown in Table 3, more than half of the respondents reported to manage their weight during MCO (84.4%). More than two-fifth of them practised a healthier eating pattern (41.2%), 36.3% reduced their physical activities, and 25.7% had a poorer sleep quality during MCO. Amongst respondents who reported having lose weight during MCO (Table 3), 68.1% claimed they managed their weight, 38.4% practised healthier eating pattern, 41.0% performed more physical activities, and 37.0% had a better sleep quality as compared to before MCO. About 29.1% of respondents who have gained weight did not manage their weight during MCO, 49.0% practised less healthy eating pattern, 38.6% performed lesser physical activities, and 38.9% had poorer sleep quality as compared to before MCO.

**Table 3:** Lifestyle behavioural changes during MCO.

Variables		Total n (%)	Weight changes during MCO			
			Decreased n (%)	No difference n (%)	Increased n (%)	*p*-value
**Lifestyle behavioural changes**						
Weight management during MCO	Never	206 (15.6)	54 (26.2)	92 (44.7)	60 (29.1)	< 0.001
	Sometimes	620 (47.0)	174 (28.1)	221 (35.6)	225 (36.3)	
	Always	493 (37.4)	197 (40.0)	176 (35.7)	120 (24.3)	
Current eating pattern compared to before MCO	Less healthy	241 (18.8)	656 (23.2)	67 (27.8)	118 (49.0)	< 0.001
	No difference	514 (40.1)	150 (29.2)	217 (42.2)	147 (28.6)	
	Healthier	528 (41.2)	203 (38.4)	194 (36.7)	131 (24.8)	
Current physical activities compared to before MCO	Never exercise	221 (16.8)	57 (25.8)	93 (42.1)	71 (32.1)	< 0.001
	Less	479 (36.3)	143 (29.9)	151 (31.5)	185 (38.6)	
	As usual	241 (18.3)	70 (29.0)	110 (45.7)	61 (25.3)	
	More	378 (28.7)	155 (41.0)	135 (35.7)	88 (23.3)	
Current sleep quality compared to before MCO	Poorer	339 (25.7)	102 (30.1)	105 (31.0)	132 (38.9)	< 0.001
	No difference	645 (48.9)	199 (30.9)	270 (41.9)	176 (27.3)	
	Better	335 (25.4)	124 (37.0)	114 (34.0)	97 (29.0)	
**Eating pattern**						
Ordering foods/ drinks through Food Delivery Apps	Never	621 (47.1)	213 (34.3)	243 (39.1)	165 (26.6)	0.017
	1–3 times/week	569 (43.1)	175 (30.8)	205 (36.0)	189 (33.2)	
	4–6 times/week	76 (5.8)	27 (35.5)	21 (27.6)	28 (36.8)	
	Everyday	53 (4.0)	10 (18.9)	20 (37.7)	23 (43.4)	
Consuming foods/ drinks from restaurants, hawker centres, coffee shops or other food stalls	Never	632 (47.9)	201 (31.8)	240 (38.0)	191 (30.2)	0.036
	1–3 times/week	484 (36.7)	175 (36.2)	172 (35.5)	137 (28.3)	
	4–6 times/week	119 (9.0)	27 (22.7)	50 (42.0)	42 (35.3)	
	Everyday	84 (6.4)	22 (26.2)	27 (32.1)	35 (41.7)	
Practicing healthier cooking methods	Never	96 (7.3)	26 (27.1)	31 (32.3)	39 (40.6)	0.002
	1–3 times/week	377 (28.6)	111 (29.4)	128 (34.0)	138 (36.6)	
	4–6 times/week	344 (26.1)	105 (30.5)	133 (38.7)	106 (30.8)	
	Everyday	502 (38.1)	183 (36.5)	197 (39.2)	122 (24.3)	
Practicing healthy eating concept “Quarter-Quarter-Half”	Never	244 (18.5)	61 (25.0)	91 (37.3)	92 (37.7)	0.002
	1–3 times/week	298 (22.6)	87 (29.2)	105 (35.2)	106 (35.6)	
	4–6 times/week	259 (19.6)	90 (34.7)	91 (35.1)	78 (30.1)	
	Everyday	518 (39.3)	187 (36.1)	202 (39.0)	129 (24.9)	
Drinking sugar-sweetened beverages	Never	486 (36.8)	169 (34.8)	190 (39.1)	127 (26.1)	0.010
	1–3 times/week	551 (41.8)	175 (31.8)	210 (38.1)	166 (30.1)	
	4–6 times/week	141 (10.7)	44 (31.2)	43 (30.5)	54 (38.3)	
	Everyday	141 (10.7)	37 (26.2)	46 (32.6)	58 (41.1)	
Consuming fried / high fat foods	Never	107 (8.1)	38 (35.5)	39 (36.4)	30 (28.0)	0.002
	1–3 times/week	492 (37.3)	172 (35.0)	194 (39.4)	126 (25.6)	
	4–6 times/week	328 (24.9)	101 (30.8)	131 (39.9)	96 (29.3)	
	Everyday	392 (29.7)	114 (29.1)	125 (31.9)	153 (39.0)	
Consuming sweet / high sugary foods	Never	173 (13.1)	69 (39.9)	67 (38.7)	37 (21.4)	0.001
	1–3 times/week	667 (50.6)	230 (34.5)	253 (37.9)	184 (27.6)	
	4–6 times/week	272 (20.6)	65 (23.9)	105 (38.6)	102 (37.5)	
	Everyday	207 (15.7)	61 (29.5)	64 (30.9)	82 (39.6)	
Consuming lunch	Never	22 (1.7)	14 (63.6)	5 (22.7)	3 (13.6)	0.003
	1–3 times/week	38 (2.9)	12 (31.6)	11 (28.9)	15 (39.5)	
	4–6 times/week	71 (5.4)	23 (32.4)	17 (23.9)	31 (43.7)	
	Everyday	1188 (90.1)	376 (31.6)	456 (38.4)	356 (30.0)	
Taking snacks	Never	158 (12.0)	60 (38.0)	53 (33.5)	45 (28.5)	0.014
	1–3 times/week	454 (34.4)	161 (35.5)	168 (37.0)	125 (27.5)	
	4–6 times/week	250 (19.0)	83 (33.2)	98 (39.2)	69 (27.6)	
	Everyday	457 (34.6)	121 (26.5)	170 (37.2)	166 (36.3)	
**Physical activity**						
Perform any physical activities ≥30 min/day	<5 days/week	1002 (76.0)	290 (28.9)	365 (36.4)	347 (34.6)	< 0.001
	≥5 days/week	317 (24.0)	135 (42.6)	124 (39.1)	58 (18.3)	
**Sleep pattern**						
Sleep latency	Good	370 (28.1)	110 (29.7)	172 (46.5)	88 (23.8)	< 0.001
	Average	426 (32.3)	137 (32.2)	151 (35.4)	138 (32.4)	
	Poor	370 (28.1)	126 (34.1)	116 (31.4)	128 (34.6)	
	Very Poor	153 (11.6)	52 (34.0)	50 (32.7)	51 (33.3)	

Eating pattern of the respondents during MCO are presented in Table 3. Overall, respondents who gained weight reported to order foods or drinks through food delivery apps (43.4% vs. 18.9%), consume foods or drinks from restaurants, hawker centres, coffee shops or other food stalls (41.7% vs. 26.2%), drank sugar-sweetened beverages (41.1% vs. 26.2), consumed fried or high fat foods (39.0% vs. 29.1%), consumed sweet or high sugary foods (39.6% vs. 29.5%), and snacking (36.3% vs. 26.5%) more frequently as compared to those who lose weight during MCO. On the other hand, respondents who lose weight tend to practise healthier cooking methods (36.5% vs. 24.3%) and comply with healthy eating concept “Quarter-Quarter-Half” (36.1% vs. 24.9%), as well as consumed lunch (31.6% vs. 30.1%) more frequently compared to those who gained weight during MCO. No significant associations were found between consumption of home-cooked meals, going out to pack foods or drinks, obtaining free foods or drinks, consumption of foods or drinks from western fast-food restaurants, baking and preparing desserts at home, consumption of rice, noodles, bread, cereals, cereal products and tubers, consumption of egg, fish, chicken, meat and meat products, consumption of legumes and nuts, consumption of milk and dairy products, consumption of fruits, consumption of vegetables, consumption of dietary supplements, consumption of probiotic drinks, as well as consumption of breakfast and dinner with body weight changes during MCO (data not shown).

In terms of physical activity, a total of 76.0% of respondents performed physical activities at least 30 minutes per day at less than five days per week during MCO. Respondents who lose weight performed physical activities at least 30 minutes per day more frequent as compared to those who gained weight (42.6% vs. 18.3%).

In terms of sleep pattern, there were more respondents had 6 to 7 hours actual sleep at night (53.7%), with average sleep latency (32.3%), and fairly good sleep quality (58.7%) during MCO. More respondents who lose weight reported to have a very poor sleep latency (34.0% vs. 33.3%) as compared to those who gained weight. There were no significant associations between duration of actual sleep at night and overall sleep quality with body weight changes during MCO (data not shown).

### Associations between lifestyle behaviours and body weight changes during MCO

Results of the multivariable generalised linear model of associations between lifestyle behaviours and body weight changes during MCO are shown in Table 4. After adjustment for confounding variables namely age, sex, ethnicity, and BMI category before MCO, practicing healthy eating concept “Quarter-Quarter-Half”, skipped lunch, and more frequent physical activities were factors accounted for significant weight loss. Respondents who never practice the healthy eating concept “Quarter-Quarter-Half” were less likely to lose weight as compared to those who practiced healthy eating concept daily (OR = 0.64, 95% CI = 0.41–0.99). Meanwhile, respondents who never consumed lunch were more likely to lose weight as compared to those with daily consumption (OR = 3.87, 95% CI = 1.27–11.73). Performing any physical activities at least 30 minutes/day for at least 5 days/week was associated with 1.4 times higher odds of weight loss among the respondents (OR = 1.44, 95% CI = 1.05–1.97).

**Table 4:** Associations between lifestyle behaviours and body weight changes during MCO.

		Weight Changes during MCO	
Variables		Decreased OR (95%CI)	Increased OR (95%CI)
Ordering foods/drinks through Food Delivery Apps	Everyday	1	1
	4–6 times/week	3.65 (1.26–10.57)	1.45 (0.56–3.72)
	1–3 times/week	1.96 (0.78–4.92)	1.08 (0.50–2.34)
	Never	2.15 (0.86–5.34)	0.82 (0.38–1.77)
Consuming foods/drinks from restaurants, hawker centres, coffee shops or other food stalls	Everyday	1	1
	4–6 times/week	0.46 (0.20–1.07)	0.68 (0.32–1.45)
	1–3 times/week	1.08 (0.53–2.23)	0.71 (0.36–1.40)
	Never	0.80 (0.39–1.63)	0.79 (0.41–1.53)
Practicing healthier cooking methods	Everyday	1	1
	4–6 times/week	0.88 (0.61–1.27)	1.14 (0.78–1.68)
	1–3 times/week	0.96 (0.65–1.43)	1.61 (1.08–2.40)*
	Never	0.97 (0.51–1.84)	1.80 (0.98–3.31)
Practicing healthy eating concept “Quarter Quarter Half”	Everyday	1	1
	4–6 times/week	1.14 (0.77–1.68)	1.24 (0.82–1.87)
	1–3 times/week	0.82 (0.55–1.22)	1.40 (0.93–2.10)
	Never	0.64 (0.41–0.99)*	1.27 (0.83–1.95)
Drinking sugar-sweetened beverages	Everyday	1	1
	4–6 times/week	1.51 (0.78–294)	1.27 (0.69–2.32)
	1–3 times/week	0.98 (0.57–1.70)	0.82 (0.49–1.36)
	Never	1.02 (0.58–1.80)	0.87 (0.51–1.47)
Consuming fried / high fat foods	Everyday	1	1
	4–6 times/week	0.92 (0.59–1.43)	0.66 (0.43–1.00)
	1–3 times/week	0.89 (0.57–1.39)	0.64 (0.41–0.99)*
	Never	1.03 (0.55–1.95)	0.89 (0.46–1.74)
Consuming sweet / high sugary foods	Everyday	1	1
	4–6 times/week	0.69 (0.40–1.20)	0.95 (0.58–1.58)
	1–3 times/week	1.05 (0.62–1.79)	0.83 (0.50–1.38)
	Never	1.17 (0.63–2.21)	0.56 (0.29–1.08)
Consuming lunch	Everyday	1	1
	4–6 times/week	1.42 (0.72–2.81)	2.39 (1.25–4.60)*
	1–3 times/week	0.99 (0.41–2.42)	1.99 (0.85–4.63)
	Never	3.87 (1.27–11.73)*	0.52 (0.11–2.45)
Taking snacks	Everyday	1	1
	4–6 times/week	1.18 (0.78–1.77)	0.70 (0.46–1.05)
	1–3 times/week	1.31 (0.92–1.88)	0.84 (0.59–1.20)
	Never	1.45 (0.88–2.38)	1.09 (0.65–1.81)
Perform any physical activities at least 30 minutes/day	<5 days /week	1	1
	≥5 days /week	1.44 (1.05–1.97)*	0.55 (0.38–0.79)*
Sleep latency	Average	1	1
	Good	0.72 (0.51–1.03)	0.62 (0.43–0.90)*
	Poor	1.26 (0.88–1.81)	1.13 (0.79–1.61)
	Very poor	1.21 (0.75–1.95)	0.54 (0.66–1.71)

Abbreviations: CI, confidence interval; OR, odds ratio.

^a^ Model was adjusted for age, sex, ethnicity, and BMI before MCO.

* *p* < 0.05.

After adjustment for confounding variables, respondents who practiced healthy cooking methods (OR = 1.61, 95% CI = 1.08–2.40) and consumed lunch (OR = 2.39, 95% CI = 1.25–4.60) less frequently were associated with higher odds of weight gain as compared to their counterparts. In contrast, respondents who consume fried/high fat foods (OR = 0.64, 95% CI = 0.41–0.99) less frequently were less likely to gain weight as compared to those with daily consumption. Performing physical activities at least 30 minutes/day for at least 5 days/week reduced the odds of weight gain by 45% (OR = 0.55, 95% CI = 0.38–0.79). In terms of sleep patterns, respondents with good sleep latency were less likely to gain weight as compared to those with average sleep latency (OR = 0.62, 95% CI = 0.43–0.90).

The associations between lifestyle behaviours and body weight changes during MCO were further analysed by adding BMI before MCO as an interaction term to the adjusted multivariable models (Table 5). Among the overweight respondents, never (OR = 4.16, 95% CI = 1.13–15.26) or less frequent practice of healthy cooking methods (OR = 2.45, 95% CI = 1.05–5.68) were associated with weight gain, omit of high fat foods were associated with higher odds of weight loss (OR = 14.98, 95% CI = 0.28–79.53), while not practising healthy eating concept were associated with lower odds of weight loss. On the other hand, obese respondents who never practiced healthy eating concept (OR = 6.32, 95% CI = 1.26–31.68) were more likely to gain weight, while those who performed physical activity more frequently were more likely to lose weight (OR = 3.35, 95% CI = 1.11–10.12). Among the normal weight respondents, those who consumed high fat foods less frequently, performed physical activity more frequently (OR = 0.53, 95% CI = 0.32–0.85), and had good sleep latency (OR = 0.52, 95% CI = 0.31–0.85) were less likely to gain weight, while those who skipped lunch were more likely to lose weight (OR = 4.76, 95% CI = 1.11–20.36). No significant associations were found between lifestyle behaviours and body weight changes during MCO among underweight respondents.

**Table 5:** Associations between lifestyle behaviours and body weight changes during MCO with interaction with BMI before MCO.

Lifestyle behaviours		Weight Changes during MCO OR (95% CI) ^a^
**Weight increased** ^**b**^		
Practicing healthier cooking methods	Everyday*overweight	1
	4–6 times/week*overweight	2.45 (1.05–5.68)*
	1–3 times/week*overweight	1.80 (0.73–4.44)
	Never*overweight	4.16 (1.13–15.26)*
Practicing healthy eating concept “Quarter-Quarter-Half”	Everyday*obesity	1
	4–6 times/week*obesity	1.41 (0.23–8.75)
	1–3 times/week*obesity	1.66 (0.41–6.63)
	Never*obesity	6.32 (1.26–31.68)*
Consuming fried / high fat foods	Everyday*overweight	1
	4–6 times/week*overweight	0.76 (0.35–1.65)
	1–3 times/week*overweight	0.46 (0.2–0.99)*
	Never*overweight	3.05 (0.49–19.05)
	Everyday*normal weight	1
	4–6 times/week*normal weight	0.46 (0.27–0.80)*
	1–3 times/week*normal weight	0.45 (0.26–0.75)*
	Never*normal weight	0.45 (0.21–0.96)*
Perform any physical activities at least 30 minutes/day	<5 days /week*normal weight	1
	≥5 days /week*normal weight	0.53 (0.32–0.85)*
Sleep latency	Average*normal weight	1
	Good*normal weight	0.52 (0.31–0.85)*
	Poor*normal weight	0.95 (0.58–1.56)
	Very poor*normal weight	0.82 (0.43–1.56)
**Weight decreased** ^**b**^		
Practicing healthy eating concept “Quarter-Quarter-Half”	Everyday*overweight	1
	4–6 times/week* overweight	0.69 (0.31–1.54)
	1–3 times/week* overweight	0.64 (0.28–1.45)
	Never* overweight	0.30 (0.11–0.84)*
Consuming fried / high fat foods	Everyday*overweight	1
	4–6 times/week*overweight	1.09 (0.50–2.37)
	1–3 times/week*overweight	1.71 (0.82–3.59)
	Never*overweight	14.98 (0.28–79.53)*
Consuming lunch	Everyday*normal weight	1
	4–6 times/week*normal weight	1.28 (0.48–3.37)
	1–3 times/week*normal weight	0.66 (0.15–2.94)
	Never*normal weight	4.76 (1.11–20.36)*
Performed physical activities at least 30 minutes/day	<5 days /week*obesity	1
	≥5 days /week*obesity	3.35 (1.11–10.12)*
Sleep latency	Average*normal weight	1
	Good*normal weight	0.53 (0.32–0.86)*
	Poor*normal weight	0.98 (0.60–1.61)
	Very poor*normal weight	0.89 (0.47–1.67)

Abbreviations: CI, confidence interval; OR, odds ratio.

^a^ Model was adjusted for age, sex, and ethnicity.

^b^ Only the significant findings were shown.

* *p* < 0.05.

## Discussion

The present study found significant changes in lifestyles behaviours among Malaysian adults during the MCO. While more respondents in our study perceived that their eating patterns were healthier during the MCO (41.2%), the overall physical activity level was reduced, with 36.3% of the adults were less active than before pandemic. On the other hand, the impact of COVID-19 pandemic lockdown on sleep patterns was equivocal, with 25.7% of the adults reported poorer and 25.4% reported better sleep quality during the MCO. The present study suggests that lifestyle behaviours including practising healthier cooking methods, daily lunch consumption, less frequent consumption of high fat foods, more frequent physical activity, and good sleep latency were associated with lower risk of weight gain, while practising healthy eating concept “Quarter-Quarter-Half”, lunch skipping, and more frequent physical activity were associated with higher odds of weight loss.

In the present study, the proportion of adults who reported having lose weight was almost similar to those who reported having gained weight. These results are contradicting with the 18.1% weight loss and 33.6% weight gain among Polish women [11] and the 12.8% weight gain among Spanish adults [14], respectively. The higher proportion of weight loss reported in our study may be explained by a high proportion of Malaysian adults who tried to manage their weight during the lockdown (37.4%) through a healthier eating pattern and maintained their workout routine at home or performed more indoor physical activities.

The present study showed that respondents who practised healthy cooking methods were less likely to gain weight during lockdown. During the lockdown, people have more time to cook at home, organise their meals, and are more likely to adopt a healthier cooking method [14]. Evidence showed that cooking at home is associated with better diet quality, lower in energy, fat, and sugar contents as compared to foods consumed away from home [26], which subsequently linked with lower prevalence of overweight and obesity [27].

We found that consumption of high fat foods was associated with higher odds of weight gain during lockdown. Early studies showed that home confinement is associated with increased consumption of unhealthy foods, especially those with high fat and sugar [13, 14, 28]. People can be stressful during home confinement due to continuously watching, reading or listening to news about COVID-19 from the media. People who are under stress may crave more for “comfort foods” that are high in sugar and fat [15–17]. High consumption or overeating of these “comfort foods” increases body weight and the risk of developing overweight and obesity [29, 30].

Although meal skipping is frequently used as a weight loss strategy, evidence showed that meal skipping, particularly breakfast skipping is associated with a higher risk of obesity [31, 32]. While no association was found between breakfast and dinner consumption with body weight changes, our study reported that lunch skipping was associated with weight gain during lockdown. As evidence explaining the associations between lunch skipping with body weight changes is lacking, it is possible that similar mechanisms underlying the association between breakfast skipping and body weight changes may be expected. Meal skipping may lead to greater hunger levels, which in turn lead to overeating at the next meal and subsequently contribute to more weight gain [33]. Meanwhile, we found that respondents who never consumed lunch were more likely to lose weight as compared to those with daily consumption. It is possible that skipping main meals may reduce total daily caloric intake and contribute to faster weight lost in short-term [34]. Meal skipping may reduce daily diet quality [34] and have a negative impact on health over time [35, 36], and should be avoided.

In year 2016, the Malaysian Healthy Plate (MHP) was created by the Ministry of Health, Malaysia to use as a visual guide for meal planning [37]. The MHP emphasises on “Quarter-Quarter-Half” concept by dividing the eating plate into three compartments, with the first quarter segment to be occupied with grain or grain products, the second quarter segment to be occupied by fish, poultry, meat, or legumes, while the third half segment includes fruits and vegetables [37]. In the present study, we found that complying to healthy eating concept “Quarter-Quarter-Half” was significantly associated with weight loss during lockdown. Practicing the QQH concept ensures a well-balanced meal comprising of a variety of food in moderate amounts and helps to maintain a healthy weight.

The Malaysian Dietary Guidelines [23] recommends that moderate intensity physical activity should be performed for at least 30 minutes on at least 5–6 days a week for maintaining good health. The present study found that adherence to the guidelines promotes weight loss and associated with lower odds of weight gain. Similar finding was reported earlier among Polish women whereby reduced physical activity during lockdown was associated with weight gain [11]. Low physical activity levels combined with unhealthy dietary behaviours during lockdown contribute to positive energy balance and weight gain. Meanwhile, our study suggests that good sleep latency was associated with lower risk of weight gain during lockdown. Lockdown during the pandemic may affect daily routine of an individual and cause stress, which in turn leads to sleep deprivation [14, 38]. Lack of sleep has been shown to alter the serum leptin and ghrelin levels, resulting in increased hunger and appetite, increased caloric intake and subsequent risk of obesity [39].

To the best of our knowledge, MyNutriLifeCOVID-19 is the first study that determines the associations between lifestyle behaviours and body weight changes among Malaysian adults during the COVID-19 pandemic lockdown. The online survey used in the present study is an ideal research tool for recruitment of large samples of survey respondents. Several limitations in the present study should be taken into consideration. First, all information including anthropometric data and lifestyle behaviours were self-reported by the respondents, which may be subjected to recall bias. Findings from the present study should be interpreted with caution as the timing of the self-reported weight was not specified. Secondly, the causal relationships between lifestyle behaviours and body weight changes could not be determined due to the cross-sectional design of the study. Thirdly, convenience sampling method used in the present study may lead to selection bias.

## Conclusions

The present study suggests that the COVID-19 pandemic lockdown changed the lifestyle behaviours of Malaysian adults, resulting in changes of body weight. Our findings could aid the development of dietary and lifestyle guidelines for a healthy weight during the pandemic. While the COVID-19 pandemic lockdown is necessary to prevent further spread of the disease, promoting healthy lifestyle practices during lockdown should be implemented for a healthy weight and better health.

## Supporting information

S1 File Dataset. (XLSX)
